# HP1β-dependent recruitment of UBF1 to irradiated chromatin occurs simultaneously with CPDs

**DOI:** 10.1186/1756-8935-7-39

**Published:** 2014-12-30

**Authors:** Lenka Stixová, Petra Sehnalová, Soňa Legartová, Jana Suchánková, Tereza Hrušková, Stanislav Kozubek, Dmitry V Sorokin, Pavel Matula, Ivan Raška, Aleš Kovařík, Jaroslav Fulneček, Eva Bártová

**Affiliations:** Academy of Sciences of the Czech Republic, Institute of Biophysics, v.v.i., Královopolská 135, 612 65 Brno, Czech Republic; Faculty of Informatics, Masaryk University, Botanická 68a, 602 00 Brno, Czech Republic; Institute of Cellular Biology and Pathology, the First Faculty of Medicine, Charles University in Prague, Albertov 4, 128 01 Prague, Czech Republic

**Keywords:** DNA-damage response, DNA repair, Irradiation, Live-cell studies, Nucleolus, UBF1

## Abstract

**Background:**

The repair of spontaneous and induced DNA lesions is a multistep process. Depending on the type of injury, damaged DNA is recognized by many proteins specifically involved in distinct DNA repair pathways.

**Results:**

We analyzed the DNA-damage response after ultraviolet A (UVA) and γ irradiation of mouse embryonic fibroblasts and focused on upstream binding factor 1 (UBF1), a key protein in the regulation of ribosomal gene transcription. We found that UBF1, but not nucleolar proteins RPA194, TCOF, or fibrillarin, was recruited to UVA-irradiated chromatin concurrently with an increase in heterochromatin protein 1β (HP1β) level. Moreover, Förster Resonance Energy Transfer (FRET) confirmed interaction between UBF1 and HP1β that was dependent on a functional chromo shadow domain of HP1β. Thus, overexpression of HP1β with a deleted chromo shadow domain had a dominant-negative effect on UBF1 recruitment to UVA-damaged chromatin. Transcription factor UBF1 also interacted directly with DNA inside the nucleolus but no interaction of UBF1 and DNA was confirmed outside the nucleolus, where UBF1 recruitment to DNA lesions appeared simultaneously with cyclobutane pyrimidine dimers; this occurrence was cell-cycle-independent.

**Conclusions:**

We propose that the simultaneous presence and interaction of UBF1 and HP1β at DNA lesions is activated by the presence of cyclobutane pyrimidine dimers and mediated by the chromo shadow domain of HP1β. This might have functional significance for nucleotide excision repair.

**Electronic supplementary material:**

The online version of this article (doi:10.1186/1756-8935-7-39) contains supplementary material, which is available to authorized users.

## Background

Genome injury by radiation or pollutants affects cellular metabolism, cell cycle, proliferation, and apoptosis, and activates DNA repair pathways. Genotoxic agents can injure DNA and induce changes in chromatin conformation. Activation of DNA repair events is associated with rearrangement of nuclear compartments, including nucleoli, nuclear bodies, and foci of accumulated proteins [1–3]. Cell-cycle control and the DNA-damage response (DDR) are also regulated by nucleolar proteins, many of which are responsible for maintaining nuclear architecture and cellular shape. For example, ultraviolet irradiation induces rearrangement of nucleolar proteins Ki-67 and WRN as well as relocation of inhibitor of growth protein 1 from the nucleoplasm to the nucleolus ( [4]; summarized by [1, 5]). Rubbi and Milner [6] suggest that the nucleolus is a stress sensor that guarantees the optimal level and nuclear distribution of p53 and that this functionality can be disrupted by genome injury. Kurki *et al.* [7] showed that ultraviolet damage induces relocation of nucleophosmin from the nucleolus to the nucleoplasm, where it interacts with p53 and HDM2 and thereby stabilizes the level of p53. These results indicate that the nucleolus is an important organelle that is sensitive to genome injury and serves unique DNA-damage-related functions. This characterization is based on the observed nucleolar protein mobility and unique repair processes of ribosomal genes [3, 8]. Moreover, Kruhlak *et al.* [9] showed that ionizing radiation inhibits RNA polymerase I (RNA pol I) activity, which substantially affects ribosomal gene transcription.

The nucleolus is a highly compartmentalized nuclear region consisting of a fibrillar center, a dense fibrillar component, and a granular component [10–12], which can separate after genome injury [13]. Nucleolar proteins, including upstream binding factor 1 (UBF1), function specifically in ribosomal biogenesis [1, 10, 14, 15]. Moreover, Moore *et al.* [3] showed that the nucleolar proteome, especially UBF1 foci, becomes highly reorganized after cell exposure to UVA irradiation [3]. The nucleolar DDR is strikingly different between ultraviolet-irradiated and γ-irradiated genomes [8], consistent with different DNA lesions that occur in response to ultraviolet and ionizing radiation. For example, cyclobutane pyrimidine dimers (CPDs) or 6-4 photoproducts, among others, are preferentially induced by UVA radiation and recognized by the nucleotide excision repair pathway [16], whereas double-stand breaks (DSBs) mostly appear as secondary lesions after γ-irradiation. The DSB-containing DNA lesions are recognized by proteins involved in non-homologous end-joining or homologous recombination repair pathways. These processes can be also initiated in ribosomal DNA because the nucleolar proteome consists of proteins involved in DSB-related repair pathways, such as ataxia telangiectasia mutated (ATM), ataxia telangiectasia and Rad3-related protein (ATR), MRE11, PARP1, and KU70/80 [3].

In this study, we investigated whether proteins involved in the DDR occupy the nucleolar UBF1-positive compartment, focusing particularly on 53BP1 and γH2AX. We also examined whether UBF1 and other selected nucleolar proteins, including fibrillarin, TCOF, RPA194, and HP1β [17, 18], co-localize with locally induced DNA lesions, which are characterized outside the nucleolus by heterochromatin protein 1β (HP1β) accumulation [19, 20]. We analyzed the distribution pattern of UBF1 at DNA lesions in live and fixed cells. Moreover, we assessed whether UBF1 recruitment to DNA lesions is cell-cycle-dependent and which DNA repair pathway is engaged in this process. Next, we examined how inhibition of ribosomal gene transcription influences UBF1 status after DNA damage, and we used Förster resonance energy transfer (FRET) to assess whether UBF1 protein binds directly to DNA or interacts with other proteins recruited to UVA-damaged chromatin. Our results show a pronounced accumulation of UBF1 at locally induced DNA lesions. Outside the nucleolus, UBF1 appeared in parallel with CPDs and interacted with accumulated HP1β at DNA lesions but did not interact with DNA. However, inside the nucleolus, UBF1 as a transcription factor binds directly to DNA. Interaction was also observed between UBF1 and HP1β, the level of which was not increased in the nucleolus after UVA irradiation. These observations suggest an existence of different DNA repair mechanisms in ribosomal genes and chromatin outside the nucleolus.

## Results

### Localization of DDR-related proteins and UBF1 in UVA-irradiated genomic regions

**Figure 1:**
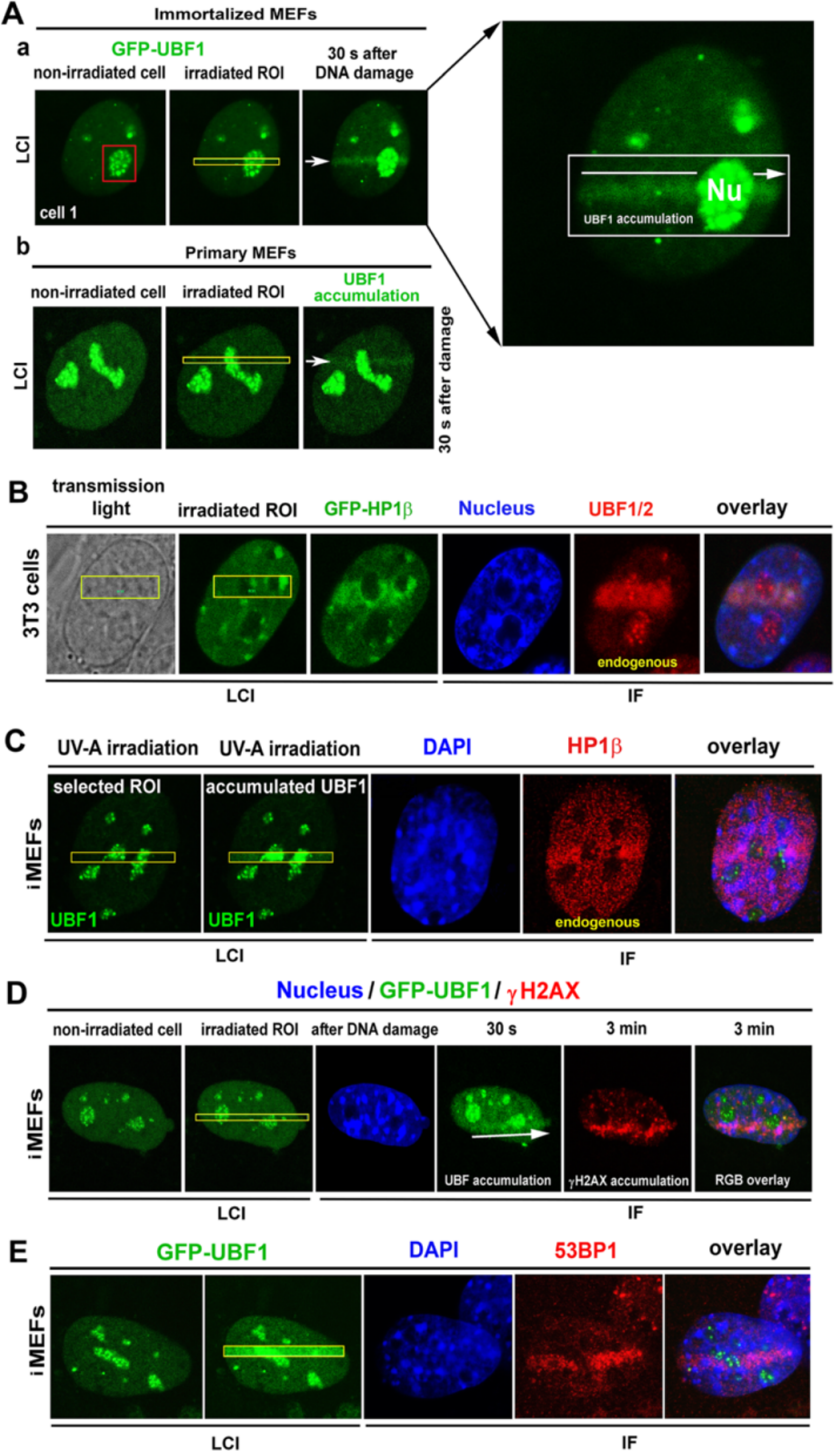
**Recruitment of GFP-UBF1, HP1β, γH2AX, and 53BP1 to UVA-induced DNA lesions. (A)** Cells transiently expressing GFP-UBF1 were microirradiated using a 355-nm UVA laser. Recombinant GFP-UBF1 (green) in **(Aa)** immortalized mouse embryonic fibroblasts and **(Ab)** primary mouse embryonic fibroblasts was monitored after local microirradiation by a 355-nm UVA laser. Magnification of the irradiated nucleus is shown. **(B)** Endogenous UBF1/2 (red) was analyzed by immunofluorescence after local UVA microirradiation of regions of interest (yellow) in 3T3 cells stably expressing GFP-HP1β (green). Irradiated cells were fixed in 4% formaldehyde and stained with appropriate antibodies against **(C)** HP1β (red), **(D)** γH2AX (red), and **(E)** 53BP1 (red). Regions of interest (yellow) were irradiated by a 355-nm UVA laser. Cell nuclei were visualized by DAPI (blue), and UBF1 was tagged by GFP (green). For each event, 10 nuclei were analyzed in three independent experiments. IF, immunofluorescence; LCI, live-cell image; Nu, nucleolus.

Using time-lapse confocal microscopy, we observed pronounced accumulation of GFP-tagged UBF1 at UVA-irradiated regions of the nucleolus and nucleoplasm (Figure 1Aa; microirradiation was performed using a 355-nm wavelength UVA laser). UBF1 recruitment to DNA lesions was observed in immortalized mouse embryonic fibroblasts (iMEFs), a non-malignant cell population that is easy to maintain in culture and test for DDR. In this experimental model, GFP-UBF1 nucleolar foci were clearly visible before irradiation (Figure 1Aa, red frame), but after local microirradiation, these small foci disappeared and the whole nucleolus became homogenously fluorescent with GFP-UBF1 (magnification in Figure 1Aa). GFP-UBF1 was also markedly recruited to DNA lesions outside the nucleolus (Figure 1Aa). Radiation-induced accumulation of GFP-UBF1 at DNA lesions was also observed in primary mouse embryonic fibroblasts (MEFs, Figure 1Ab). We verified recruitment of UBF1 using an appropriate antibody and found elevated levels of endogenous UBF1/2 at DNA lesions in parallel with an increase in full-length GFP-HP1β protein levels (Figure 1B). We also confirmed the accumulation of endogenous HP1β at UVA-damaged chromatin (Figure 1C, [19, 20]). To verify genome injury by local microirradiation, we confirmed γH2AX- and 53BP1-positivity at DNA lesions using specific antibodies (Figure 1D,E). After irradiation of nucleoli, high levels of 53BP1 and γH2AX were observed in the area surrounding UBF1-positive nucleolar regions, which were characterized by an increase in UBF1 after irradiation (Figure 1D,E). Similar to 53BP1 and γH2AX, we also observed a pronounced accumulation of HP1β, particularly at UVA-damaged regions around or outside the nucleolus (Figure 1B,C).

However, we recently showed that HP1β not only occupies chromatin outside the nucleolus, but subtle amount of HP1β can be also detected in ribosomal genes by chromatin immunoprecipitation PCR [18]. Using the same experimental approach, we also observed a high level of γH2AX at ribosomal genes of non-irradiated cells [8]. Therefore, in this study, we not only analyzed UBF1 as an important nucleolar protein but also studied additional nucleolar proteins, including HP1β and γH2AX, and their potential to be recruited to nucleolar DNA lesions. We also studied the presence of RNA pol I subunit RPA194, TCOF, and fibrillarin at DNA lesions (Additional file 1: Figure S1) by subjecting cell nuclei to local microirradiation. DNA lesions were identified by markedly increased GFP-HP1β fluorescence, but RPA194, TCOF, and fibrillarin were not recruited to UVA-damaged chromatin (compare irradiated cells in Additional file 1: Figure S1 with non-irradiated cells in Additional file 2: Figure S2Aa-d).

### UBF1 status at DNA lesions with respect to cell cycle phases and DNA repair pathways

**Figure 2:**
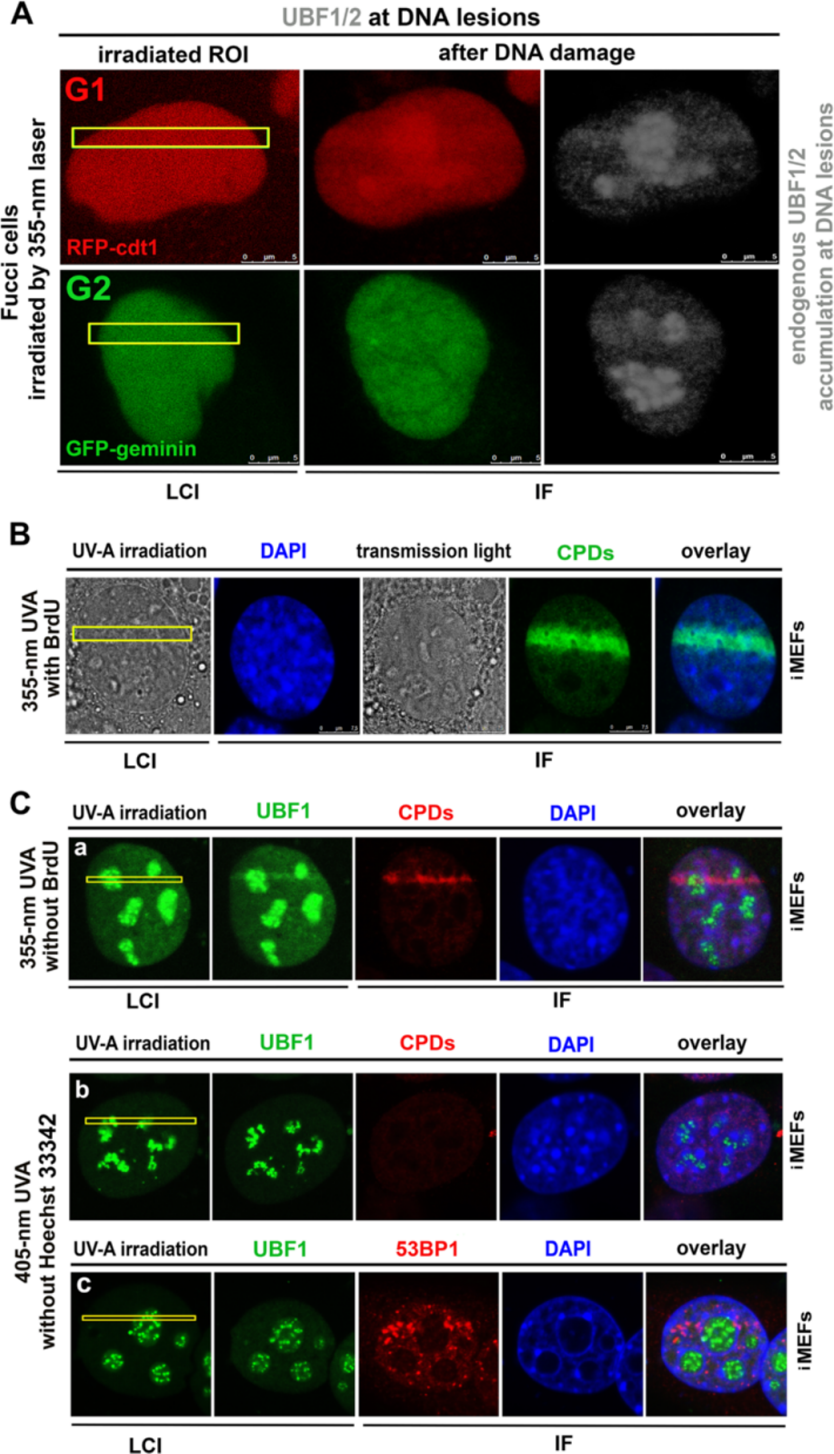
**UBF1/2 recruitment to DNA lesions in G1 and G2 cell-cycle phases and involvement in DNA repair pathways. (A)** Recruitment of endogenous UBF1/2 (black-and-white mode) to locally induced DNA lesions (yellow frames) in a HeLa-Fucci cellular model was observed in both G1 (red, expression of RFP-cdt1) and G2 (green, expression of GFP-Geminin) cell cycle phases. **(B)** CPDs (green) induced by a 355-nm UVA laser with BrdU pre-sensitization. Nuclei of live cells (LCI) were visualized by transmited light microscopy (grey) and nuclei of fixed cells were stained with DAPI (blue). **(C)** Cells were irradiated by a 355-nm UVA laser without BrdU pre-sensitization, and **(Ca)** GFP-UBF1 (green) and CPDs (red) appeared in regions of interest. When cells were irradiated by a 405-nm UVA laser, **(Cb)** CPDs (red) were not detected in regions of interest. **(Cc)** GFP-UBF1 (green) was not recruited to regions of interest after irradiation by a 405-nm UVA laser, but 53BP1 (red) accumulated at irradiated regions of interest (yellow). For each event, 10 nuclei were analyzed in three independent experiments. IF, immunofluorescence; LCI, live-cell image.

We analyzed the accumulation of UBF1 at DNA lesions in HeLa cells expressing fluorescent ubiquitination-based cell-cycle indicators (Fucci). We were able to distinguish G1 and G2 phases in a HeLa-Fucci cellular model, which stably expresses RFP*-*Cdt1 during the G1 phase and GFP-Geminin during the G2 phase [21]. These results show that UBF1 recruitment to chromatin at DNA lesions was independent of cell cycle phase (Figure 2A).

Next, we analyzed UBF1 involvement in DNA repair pathways. We used a UVA laser (355-nm wavelength) to induce DSBs, which are recognized by non-homologous end-joining or homologous recombination repair mechanisms. To confirm the induction of DSBs, we analyzed γH2AX- and 53PB1-positivity in irradiated regions (Figure 1D,E, [20, 22]). Additionally, we tested possible activation of the nucleotide excision repair pathway by a UVA laser (355-nm wavelength, either with or without 5-bromo-2′-deoxy-uridine (BrdU) sensitization; see Methods section) and observed an increased appearance of CPDs (Figure 2B,Ca). We also used a 405-nm UVA laser to specifically induce γH2AX- or 53BP1-positive DSBs but not CPDs ( [22], Figure 2Cb,Cc). The results of this experiment show that UBF1 was only recruited to chromatin with CPDs, suggesting that UBF1 is involved in the nucleotide excision repair pathway.

### UBF1 status at DNA lesions after suppression of transcription

**Figure 3:**
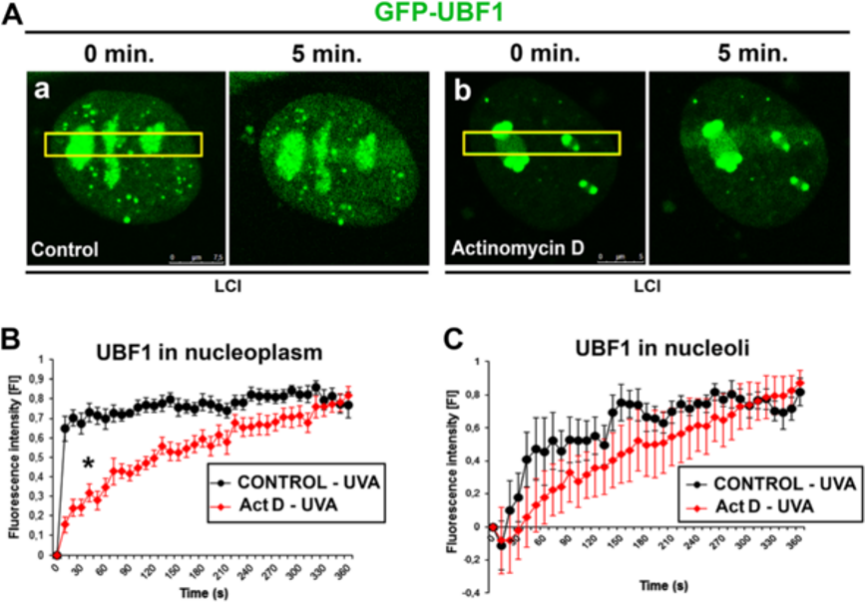
**Fluorescence intensity of GFP-UBF1 after UVA irradiation in non-treated and actinomycin D-treated cells. (A)** Recruitment of GFP-UBF1 to UVA-induced DNA lesions in regions of interest (yellow) of **(a)** non-treated cells and **(b)** actinomycin D-treated cells. Images show normalized intensity of GFP-UBF1 fluorescence in UVA-induced DNA lesions in iMEFs treated with actinomycin D and non-treated iMEFs. Fluorescence intensity was measured for GFP-UBF1 in **(B)** irradiated nucleoplasm and **(C)** irradiated nucleoli. Approximately 40 nuclei were analyzed for each control and actinomycin D-treated cells. LCI, live-cell image.

We next examined whether UBF1 recruitment to DNA lesions is affected by suppression of transcription-related processes by testing whether inhibition of RNA polymerases abrogates UBF1 recruitment to UVA-irradiated chromatin. We anticipated that UBF1 recruitment to DNA lesions would be antagonized by actinomycin D treatment, but this was not observed in the nucleolar compartment (Figure 3A-C; see Figure 3C in particular). GFP-UBF1 was recruited to DNA lesions in both non-treated control cells (Figure 3Aa) and actinomycin D-treated cells (Figure 3Ab). Actinomycin D treatment delayed UBF1 accumulation only at UVA-induced DNA lesions in the nucleoplasm (Figure 3B). Outside the nucleolus, UBF1 was recruited to DNA lesions in non-treated cells 15 s after irradiation, whereas the level of UBF1 recruited to DNA lesions in actinomycin D-treated cells was similar to that in non-treated cells as late as 5 min after UVA irradiation. This delay was statistically significant for UBF1 accumulation in irradiated nucleoplasm (Figure 3B) but not in nucleoli (Figure 3C).

### UBF1 interaction with selected DDR-related proteins

**Figure 4:**
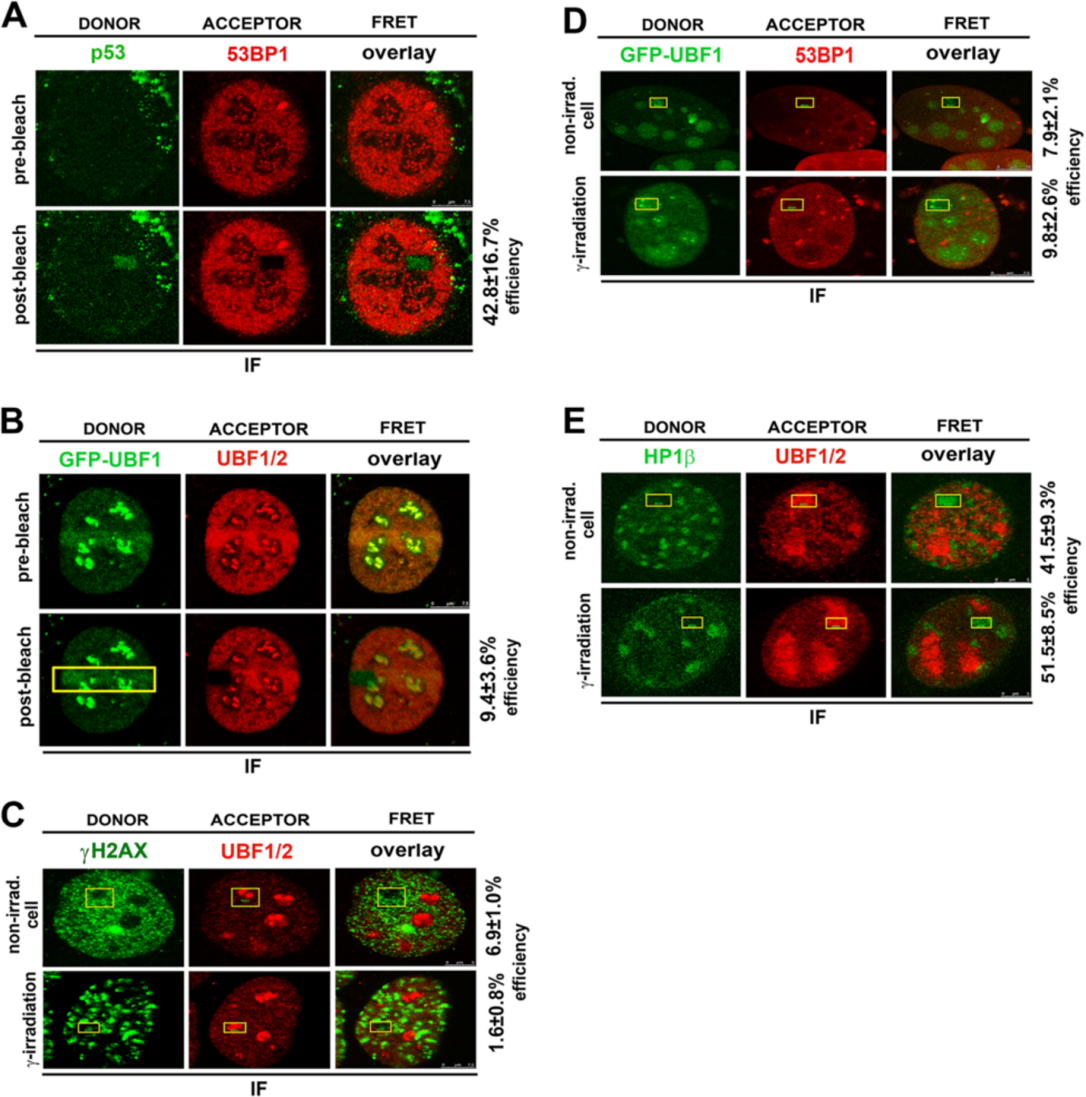
**Protein interactions at DNA lesions. (A)** FRET results for reference interacting partners p53 (green) and 53BP1 (red). **(B)** FRET analysis of UBF1/2 dimerization. FRET analysis of **(C)** UBF1/2 (red) and γH2AX (green), **(D)** GFP-UBF1 (green) and 53BP1 (red), and **(E)** HP1β (green) and UBF1/2 (red). Studies were performed in control and γ-irradiated cells using a Leica TSC SP5 confocal microscope. For each event, 10 nuclei were analyzed in three independent experiments. IF, immunofluorescence; non-irrad., non-irradiated cells.

**Figure 5:**
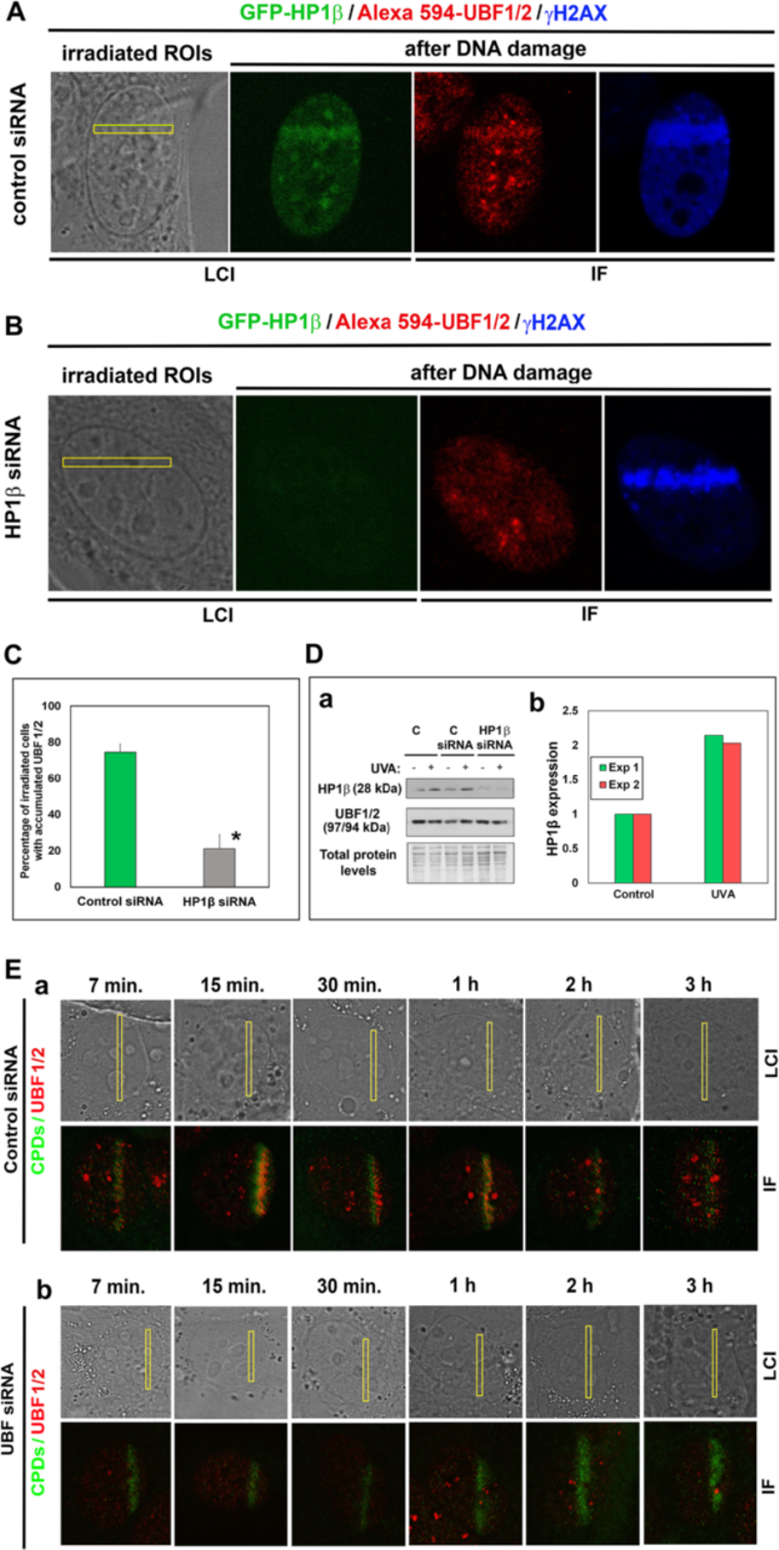
**siRNA silencing of HP1β reduced UBF1/2 accumulation after UVA irradiation. (A)** Recruitment of endogenous UBF1/2 (red) to locally induced, γH2AX-positive (blue) DNA lesions (yellow frames) in 3T3 cells stably expressing GFP-HP1β (green) in control sample relevant to siRNA experiments. **(B)** Effect of HP1β siRNA on UBF1/2 (red) accumulation after UVA irradiation by a 355-nm UVA laser. Induced DNA lesions were positive for γH2AX (blue). **(C)** Percentage of irradiated cells with accumulated UBF1/2. Thirty cell nuclei were evaluated for each event. Asterisks show statistically significant differences from control values (*P* ≤ 0.05) using Student’s *t* tests. **(D)** Efficiency of HP1β siRNA was determined by **(a)** Western blots showing the level of HP1β and UBF1/2. Samples were loaded according to identical total protein levels. Results of **(b)** qRT-PCR experiments show up-regulation of HP1β in UVA-irradiated cells. Results were obtained from two biological replicates. The transcripts were normalized to two housekeeping reference genes (*GAPDH* and *β-actin*). Irradiation was performed by UVA lamp **(E)** Analysis of CPD accumulation at DNA lesions in **(a)** control cells and **(b)** cells exposed to UBF siRNA. Image acquisition was performed 7 min, 15 min, 30 min, 1 h, 2 h, and 3 h after local microirradiation by a 355-nm UVA laser. For panel E, 40 nuclei were examined. C, control; Exp 1, first independent experiment; Exp 2, second independent experiment; IF, immunofluorescence; LCI, live-cell image.

We observed pronounced and rapid accumulation of UBF1 at locally induced DNA lesions (Figure 1A,B). Therefore, as a next step, we analyzed the functional significance of this phenomenon by performing FRET analysis to investigate potential interactions between UBF1 and 53BP1, UBF1 and γH2AX, or UBF1 and HP1β. We also analyzed potential UBF1 dimerization at DNA lesions (Figure 4). To optimize FRET analysis, we used the reference interacting partners 53BP1 and p53 in a non-irradiated genome and observed a FRET efficiency of 42.8 ± 16.7% using our microscopy system (Figure 4A). The FRET efficiency of GFP-UBF1 and Alexa 594-UBF1 was 9.4 ± 3.6% at DNA lesions (Figure 4B) and 12.9 ± 9.1% in non-irradiated nucleoplasm (not shown). These results indicate that UBF1 does not form dimers at DNA lesions. Similarly, the FRET efficiency was 1.6 ± 0.8% for UBF1 and γH2AX and 9.8 ± 2.6% for UBF1 and 53BP1 in the genome irradiated with 5 Gy of γ-rays (Figure 4C,D). In the non-irradiated genome, the FRET efficiency for these proteins was 6.9 ± 1.0% and 7.9 ± 2.1%, respectively (Figure 4C,D). This indicates that UBF1 does not interact with γH2AX or 53BP1 at DNA lesions. However, FRET analyses showed that UBF1 and HP1β interact with a high probability in the nucleolus in non-irradiated and γ-irradiated regions, with efficiencies of 41.5 ± 9.3% and 51.5 ± 8.5%, respectively (Figure 4E). The FRET efficiency for UBF1 and HP1β at the nucleus was 33.6 ± 10.9% (not shown). These results suggest that UBF1 is recruited to DNA lesions because of its interaction with HP1β. Therefore, we tested the effect of HP1β siRNA on UBF1 recruitment to γH2AX-positive DNA lesions (Figure 5A-C). Whereas control cells displayed markedly increased HP1β levels and UBF1 recruitment to DNA lesions (Figure 5A), HP1β siRNA reduced the level of cellular HP1β and resulted in no or subtle recruitment of UBF1 to γH2AX-positive DNA lesions (Figure 5B; quantification shown in Figure 5C). We confirmed the efficiency of HP1β siRNA by performing Western blot analysis, which showed that RNA interference reduced the level of HP1β (Figure 5Da). Interestingly, UVA irradiation of control cells increased HP1β levels, as shown by Western blots (Figure 5Da), and qRT-PCR showed up-regulation of HP1β (Figure 5Db).

After knock-down of UBF1, we also found a delay in the appearance of CPDs from 7 min to 3 h after UVA irradiation (compare control in Figure 5Ea with UBF siRNA in Figure 5Eb). These experiments confirm the importance of UBF1 in the nucleotide excision repair pathway.

### Overexpression of HP1βΔCSD, but not HP1βΔhinge or HP1βΔCD, has a dominant-negative effect on UBF1 recruitment to DNA lesions

**Figure 6:**
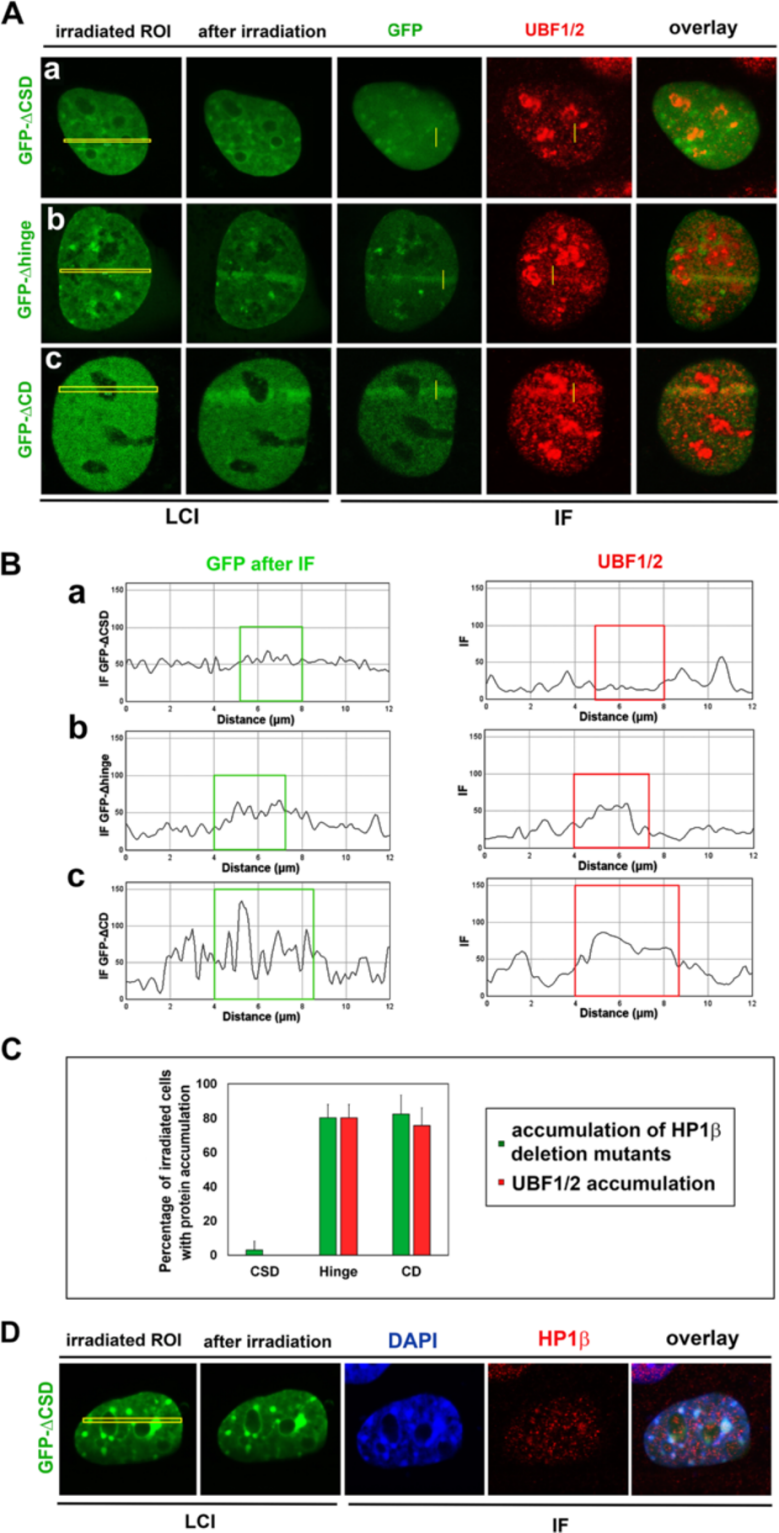
**Functional analysis of HP1βΔCSD, HP1βΔhinge, and HP1βΔCD for UBF1 recruitment to UVA-induced DNA lesions. (A)** Analysis of endogenous UBF1/2 (red) recruitment to UVA-induced DNA lesions (yellow regions of interest) in MEFs transiently expressing **(a)** GFP-HP1β-ΔCSD, **(b)** GFP-HP1β-Δhinge, or **(c)** GFP-HP1β-ΔCD (all green). **(B)** Quantification of fluorescence intensity of UBF1/2 (red) and HP1β (green) from panel A. Quantification was performed for **(a)** GFP-HP1β-ΔCSD, **(b)** GFP-HP1β-Δhinge, and **(c)** GFP-HP1β-ΔCD (all green). **(C)** Percentage of cells with recruitment of studied proteins to DNA lesions. Thirty cells were analyzed for each event in three independent experiments. **(D)** Analysis of endogenous HP1β (red) at UVA-induced DNA lesions (yellow regions of interest ) in mouse embryonic fibroblasts transiently expressing GFP-HP1β-ΔCSD (green). For panel D, 20 nuclei were analyzed. CD, chromodomain; CSD, chromo shadow domain; IF, immunofluorescence; LCI, live-cell image.

**Figure 7:**
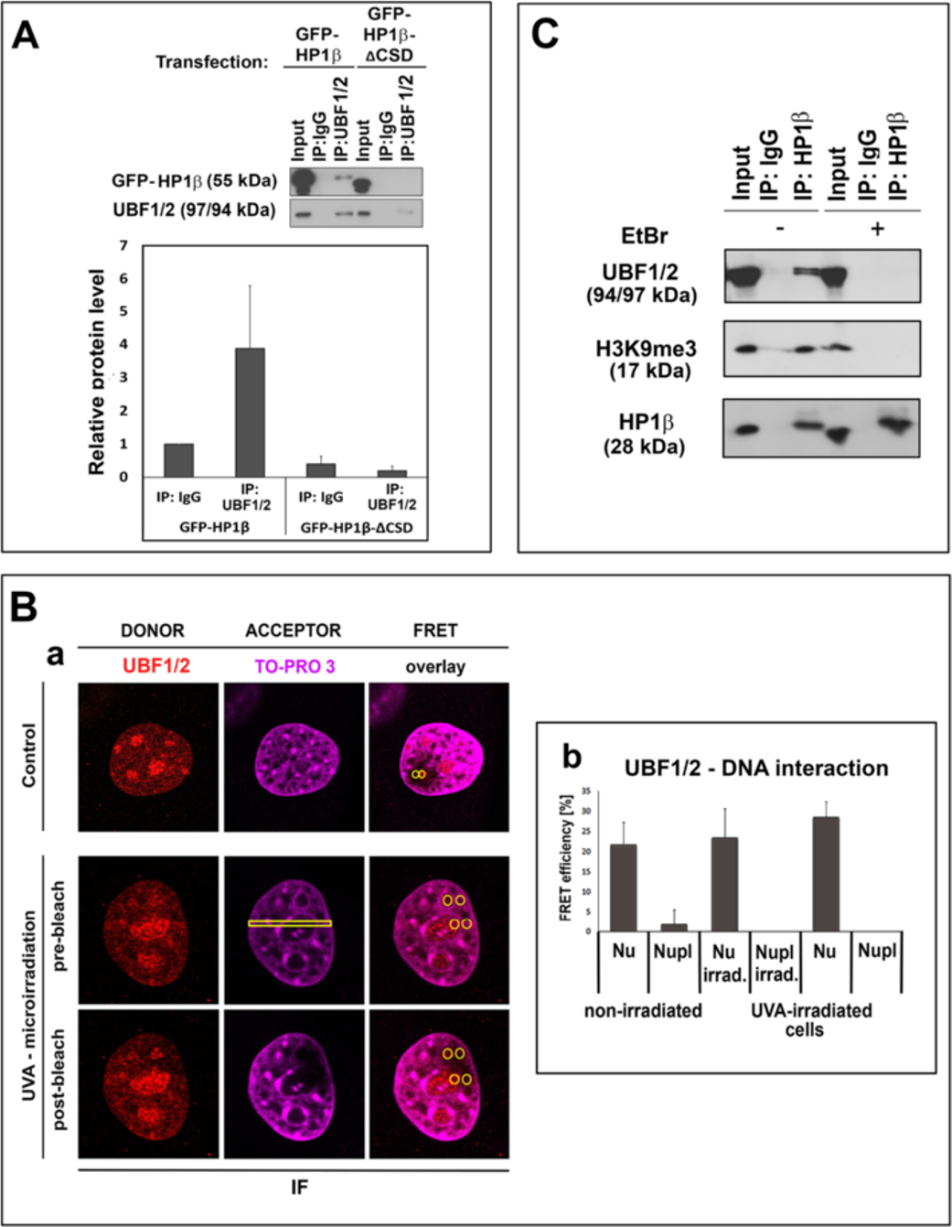
**Interaction of UBF1 with UVA-damaged chromatin. (A)** Cells expressing full-length GFP-HP1β and GFP-HP1β-ΔCSD were immunoprecipitated with antibody against UBF1/2, and subsequent Western blot analysis of HP1β was performed. Quantification of immunoprecipitation fragments and original Western blots are shown. **(B)** FRET analysis of potential interactions between UBF1/2 (Alexa 594, red) and DNA (stained by TO-PRO-3, far-red, 642/661 nm). **(Ba)** Regions of interest (yellow) inspected by FRET; **(Bb)** quantification of UBF1/2 interaction with DNA in the nucleolus and nucleoplasm of irradiated and non-irradiated cells. FRET efficiency is shown in percentage ± standard error. For each event, 10 nuclei were analyzed in three independent experiments. **(C)** Immunoprecipitation verified the interaction between HP1β and UBF1/2; HP1β and H3K9me3 were used as reference interacting partners. Immunoprecipitation was performed in the presence or absence of EtBr; 20 μg of protein lysate was used as input. IF, immunofluorescence; irrad., irradiated; Nu, nucleolus; Nupl, nucleoplasm.

We tested whether deletion of the HP1β chromodomain (CD), hinge region, and chromo shadow domain (CSD) affects recruitment of endogenous UBF1 to DNA lesions (Figure 6A, B). Using iMEFs transiently expressing GFP-HP1β-ΔCSD, we confirmed that this recombinant protein did not significantly accumulate at DNA lesions, as previously reported by Luijsterburg *et al.* [20]. We also observed that endogenous UBF1 was not recruited to DNA lesions with transient expression of GFP-HP1β-ΔCSD (Figure 6Aa,Ba). By contrast, deletion mutants of HP1β lacking the CD or hinge region were recruited to UVA-damaged sites, similar to full-length HP1β (Figure 6Ab,Ac,Bb,Bc). Deletions in the HP1β CD and hinge region did not affect recruitment of endogenous UBF1 to locally induced DNA lesions (Figure 6Ab,Ac,Bb,Bc; quantification of the whole cell population is shown in Figure 6C). Although we cannot exclude the possibility that UBF1 interacts with endogenous HP1β in cells overexpressing GFP-HP1β-ΔCSD, no increase in endogenous HP1β was detected at UVA-induced DNA lesions of these cells (Figure 6D). For further clarification, cells overexpressing full-length GFP-HP1β or GFP-HP1β-ΔCSD were immunoprecipitated with antibody against UBF1. Subsequent Western blot analysis of HP1β detected no interaction between GFP-HP1β-ΔCSD and endogenous UBF1, but a potential interaction between GFP-HP1β and endogenous UBF1 was detected (Figure 7A).

### UBF1 binds to DNA in only nucleolus

FRET analysis showed that UBF1 binds to TO-PRO-3-stained DNA in the nucleolus but not to non-ribosomal DNA (Figure 7Ba,Bb). These experiments demonstrate that UBF1 functions as a transcription factor in ribosomal genes because of its binding to DNA in the nucleolus. This was observed in the non-irradiated and UVA-irradiated genome (see quantification in Figure 7Bb). FRET analysis not only showed that UBF1 interacted with HP1β (Figure 4E) but also confirmed UBF1 binding to ribosomal DNA (Figure 7Ba,Bb). These results also serve as verification of our FRET analyses.

Next, we examined the interaction of UBF1 and HP1β in entire nucleus using immunoprecipitation (Figure 7C). By this experimental technique, we confirmed the interaction between HP1β and UBF1 (reference interacting partners were HP1β and H3K9me3). Moreover, in the presence of ethidium bromide (EtBr), which protects DNA from binding to protein complexes, we did not observe an interaction between HP1β and UBF1. This event should be related to ribosomal genes, which are occupied by UBF1. Therefore, UBF1 protein-DNA and UBF1-HP1β interactions might play a role not only in the transcription of ribosomal genes but also in DDR-related events in the nucleolus ( [18]; Figures 4E, and 7B,C).

Together, our data show that UBF1 is recruited to DNA lesions, owing to its interaction with HP1β. This DDR-related event is likely dependent on a functional CSD of HP1β. Moreover, as expected in the nucleolus, the transcription factor UBF1 binds directly to DNA. From a functional point of view, it is possible that radiation induces UBF1 release from promoter regions of transcribed genes. Therefore, UBF1 may accumulate at sites of DNA damage because of an affinity for altered chromatin topologies.

Our findings indicate that UBF1 recruitment to DNA lesions is a unique DDR of this transcription factor and not an artifact of GFP tagging or transient transfection. This conclusion is based on results of immunofluorescence staining of endogenous UBF1, which co-localized with DNA lesions (Figure 1B). Moreover, Western blot analysis detected a weaker signal for recombinant UBF1 than for endogenous UBF1 (Additional file 2: Figure S2B). This shows that our transient cell transfection for UBF1 visualization by GFP was successfully optimized and did not induce apoptosis, as we detected no lamin B fragmentation (Additional file 2: Figure S2B).

## Discussion

In this study, we investigated genome responses to ultraviolet and γ-radiation and accumulation patterns of UBF1 and HP1β at UVA-damaged chromatin in live cells. Radiation is considered a genotoxic agent that induces genomic DNA lesions accompanied by changes in nuclear and nucleolar morphologies. Different types of radiation can cause nucleolar segregation, nuclear relocation of proteins, and changes in protein post-translational modifications [4, 6, 8, 23–28]. Ionizing radiation can induce nucleolar disruption, leading to ATM-dependent inhibition of RNA pol I activity, thereby suppressing ribosomal gene transcription [6, 9]. A similar silencing effect is ascribed to actinomycin D, which primarily inhibits RNA pol I activity and induces dose-dependent single-strand breaks [29, 30]. Here, actinomycin D delayed UBF1 recruitment to nucleoplasmic DNA lesions (Figure 3B).

Our data are consistent with those of Moore *et al.* [3], who observed radiation-induced rearrangement of UBF1-positive nucleolar regions, and demonstrate that some nucleolar proteins, including UBF1, respond to DNA injury and relocate in a damage-specific manner (Figures 1A and 2B,C). Here, we show for the first time the direct recruitment of endogenous and recombinant UBF1 to UVA-damaged chromatin (Figure 1A,B). We determined that UBF1 but no other nucleolar proteins, such as RPA194, TCOF, or fibrillarin co-localized with locally induced DNA lesions (Figure 1A,B, and Additional file 1: Figure S1A-C). UBF1 accumulated at DNA lesions in G1 and G2 cell-cycle phases, and interacted with other DNA-damage-related proteins, such as HP1β (Figures 1B, 2A, and 4E). Moreover, overexpression of HP1βΔCSD had a dominant-negative effect on UBF1 recruitment to DNA lesions (Figure 6A-C).

Our results are also consistent with those of Luijsterburg *et al.* [20], who showed the importance of CSD for HP1β recruitment to DNA lesions. Here, we additionally specified HP1β functioning at DNA lesions by its interaction with UBF1 (Figures 1B, 4E, and 6A-C). Moreover, we showed that recruitment and interaction of HP1β with UBF1 at DNA lesions is a specific event appearing simultaneously with CPDs (Figure 2C), recognized by the nucleotide excision repair pathway [31, 32]. This process is not accompanied by UBF1 dimerization at DNA lesions (Figure 4B). Moreover, UBF1, as a transcription factor for ribosomal genes, interacts with DNA only in the nucleolus but not outside nucleolar regions in non-irradiated and UVA-irradiated cells (Figure 7Ba,Bb).

Emerging evidence indicates that HP1 isoforms have a role in the DDR [33] and here we showed the same for UBF1. It is well known that HP1 isoforms can be recruited to ultraviolet-induced DNA lesions, oxidative lesions, and ionizing radiation-induced DNA breaks. Luijsterburg *et al.* [20] showed that HP1 homologues are recruited to UVA-damaged chromatin, positive on CPDs, simultaneously with γH2AX- and 53BP1-positive DSBs. Similar results were found in the present study for CPDs, 53BP1, and γH2AX when we used irradiation by 355-nm UVA laser (Figures 1D,E and 2B). However, 405-nm UVA laser irradiation did not induce CPDs, but only 53BP1 (Figure 2Cc) or γH2AX positivity [22]. This experimental approach revealed an association of UBF1-HP1β with only CPDs, but not with DSBs. Interestingly, Zarebski *et al.* [34] documented the appearance of HP1 isoforms at sites of oxidative damage together with OGG1 and XRCC1, which are factors involved in base excision repair. According to these findings, it seems evident that the recruitment of HP1β, and possibly UBF1, should be assessed in a manner sensitive to the complexity of several simultaneous DNA repair pathways, as suggested by Dinant and Luijsterburg [33].

As UBF1 is a ribosomal gene transcription factor, the theory of transcription-coupled repair (TCR) (summarized by [31, 35]) is relevant to our investigations. We showed that UBF1 colocalizes with CPDs, which are recognized by the nucleotide excision repair pathway, and it is well known that TCR is a variant of nucleotide excision repair [31]. The TCR pathway is associated with protein-coding genes, but we observed pronounced UBF1 recruitment in the nucleolus, where ribosomal genes reside (see detail of UBF1 and nucleoli in Figures 1A,B and 3C). The TCR model assumes that RNA pol II is arrested when the transcriptional machinery recognizes DNA lesions. This stalling of the massive transcription apparatus effectively signals to DNA repair proteins through the Cockayne syndrome B or A proteins [35], which also function during ribosomal gene transcription [36]. Thus, DDR and ribosomal gene transcription converge at this point. In the present study, we also must consider the possibility that DSBs might be recognized by signaling pathways other than DNA repair pathways. For example, Ju *et al.* [37] and Haffner *et al.* [38] report that strand breaks may be a part of other genomic processes, including transcription. In some cancer cells, the transcriptional program of fundamental genes involves the formation of DSBs, and recruitment of DSB-related repair proteins can appear in parallel with the relocation of chromatin loops of activated genes to transcription sites, called transcription potentially factories. This transcription-related event requires topoisomerase II β activity, which is responsible for DNA coiling [38] (summarized by [39]). Moreover, our results allow the possibility that other transcription factors can be also recruited to DNA lesions (Figure 1A,B; [40]).

## Conclusions

The results of our study indicate that the nucleolus and the nucleolar UBF1 protein are important components of the cellular response to genome injury [1, 41]. The phenomenon of DNA-damage-induced nucleolar segregation is well documented [23, 42]. Thus, the responses of nucleolar proteins to radiation and radiation-induced changes in nucleolar morphology should be an area of study with respect to tumor radiotherapy. Our data show that the nucleolar UBF1 protein interacts with HP1β, and that both proteins co-localize with CPDs. This radiation-induced event can be influenced by overexpression of HP1βΔCSD, which has a dominant-negative effect on UBF1 recruitment to DNA lesions, and this DDR could be associated with extensive chromatin conformational changes induced by UVA irradiation [43]. Together, our results indicate that UBF1 recruitment to DNA lesions probably depends on CPDs and the presence of HP1β at UVA-damaged chromatin, as shown by immunofluorescence, FRET analysis, and siRNA experiments (Figures 2C, 4E, and 5B,C).

## Methods

### Cell cultivation and transfection

The iMEFs were a generous gift from the laboratory of Professor Thomas Jenuwein (Max Planck Institute of Immunobiology and Epigenetics, Freiburg, Germany). These cells were used as wild-type controls and were cultivated in DMEM with 10% FBS and appropriate antibiotics at 37°C in a humidified atmosphere containing 5% CO_2_. The iMEFs were immortalized according to the following standard protocol: every third day, the cell culture was split and counted, and 3 × 10^5^ cells were transferred to a new 10-cm plate. Immortalization occurred through natural selection; cell irradiation was not used. The results obtained from iMEFs were verified in primary MEFs isolated from 12.5-day embryos of ICR mice according to the method of Bártová *et al.* [44]. ICR mice were obtained from the Breeding Facility of the Medical Faculty, Masaryk University, Brno, Czech Republic. The MEF isolation was performed according to an experimental protocol confirmed by the National and Institutional Ethics Committee (protocol No. 224/2012). Mice were kept under standard conditions, and their use followed the European Community Guidelines of accepted principles for the use of experimental animals. Mice were sacrificed by overexposure to anesthetics (Narcamon/Rometar solution, Spofa, Czech Republic). For additional analysis, we used 3T3 cells stably expressing GFP-HP1β (a generous gift from Dr. Paul Verbruggen, Swammerdam Institute for Life Sciences, University of Amsterdam, Netherlands). Dr. Martijn S. Luijsterburg (Department of Cell and Molecular Biology, Karolinska Institute, Stockholm, Sweden) generously provided plasmids encoding GFP-HP1β-ΔCSD, GFP-HP1β-Δhinge, and GFP-HP1β-ΔCD [20]. For studies on cell-cycle-dependent recruitment of UBF1 proteins to DNA lesions, we used HeLa-Fucci cells expressing RFP*-*Cdt1 (red fluorescence) in the G1 phase and GFP-Geminin (green fluorescence) in the G2 phase [21].

For selected experiments, cells were treated with 0.5 μg/ml actinomycin D (#A9415, Sigma-Aldrich) for 2 h. For transfection of iMEFs, cells were cultivated on glass-bottomed tissue culture dishes to 70% confluence, and transfected with 2 to 5 μg plasmid DNA encoding GFP-UBF1 (#17656; Addgene, Cambridge MA, USA), GFP-p53 (#12091; Addgene, Cambridge MA, USA). Transfections were performed using METAFECTENE™PRO reagent (#T040-2.0, Biontex Laboratories GmbH, Germany). These methodological parameters were used for all cell transfection experiments.

### Induction of DNA lesions and confocal microscopy

For local microirradiation using a UVA laser (355-nm or 405-nm wavelength), cells were seeded on uncoated, γ-irradiated, 50-mm glass-bottomed dishes used for inverted microscopy (No. 0; MatTek Corporation, USA, #P50G-0-30-F) or 35-mm grid-500 μ-dishes (#81166, Ibidi, Germany). At 70% confluence, the cells were sensitized with BrdU according to the method of Šustáčková *et al.* [45] and subsequently UVA irradiated. BrdU incorporation was performed to increase the radiosensitivity of cells [46]. These experiments were performed using a previously described protocol [20]. Briefly, cells were sensitized with 10 μM BrdU for 16 to 18 h before local microirradiation using a 355-nm UVA laser (all figures except Figure 2Ca-c). To control BrdU incorporation, cells were stained using the BrdU labeling and detection kit I (#11296736001, Roche, Prague, CZ) (not shown). We also examined DDR events without BrdU sensitization [40, 47] and observed low-level GFP-UBF1 recruited to sites of DNA damage (Figure 2Ca) similar to that described by Bártová *et al.* [40] for GFP-OCT4 recruitment to DNA lesions. Irradiation using a 405-nm UVA laser was performed here without pre-sensitization (Figure 2Cb,Cc). Usually, cell treatment by Hoechst 33342 (Invitrogen) for 5 min is applied before irradiation by a 405-nm UVA laser [19, 20], but we did not apply this experimental step, in order to be closer to the induction of a single DNA repair pathway. Another experimental approach [47] is to apply different intensities of a 405-nm laser for cell irradiation. This was not used here because our laser setting was optimal for our analyses.

In all cases, in the present experiments, defined regions of interest (ROIs) in the nucleolus and outside the nucleolar region were irradiated by 100% laser power for 4 s. Laser intensity was not reduced at the acousto-optic tunable filter. No protein accumulation at UVA-damaged chromatin was considered when fluorescence intensity was identical at irradiated and non-irradiated regions. We considered protein accumulation as a fluorescence intensity higher than 30% of standard cellular level.

The UVA irradiation was performed using a Leica SP5 X confocal microscope at the following settings: 512 × 512 pixels, 400 Hz, bidirectional mode, 64 lines, zoom 8 to 12. We used the following settings for confocal scanning: 1024 × 1024 pixels, 400 Hz, bidirectional mode, 4 lines, zoom 8 to 12. Before immunofluorescence staining, live cells were monitored in real time, and ROIs were selected and irradiated. When increased levels of proteins of interest appeared, cells were fixed in 4% formaldehyde for 10 min, and immunostaining was performed. We irradiated approximately 10 nuclei per microscope dish (for each experiment, we used at least three dishes), and the whole procedure took approximately 30 min. After immunodetection, locally irradiated cells were monitored according to defined coordinates marked on the microscope dishes.

In experiments involving γ-irradiation of the whole cell population, cells were irradiated with 5 Gy of γ-rays using a ^60^Co source, and analyses were performed 2 h after γ-irradiation. No pre-sensitization was used.

### Specification of radiation sources

#### UVA laser, 355 nm

Laser from Coherent, Inc.; laser power, 80 mW; irradiated area, 25.8 × 10^-8^ cm^2^; irradiation time, 4 s; resolution for image acquisition, 512 × 512; line average, 64; pixel size, 60.06 × 60.06 nm; image size, 30.74 × 30.74 μm; total number of irradiated pixels, 24,455; irradiation time per pixel, 122 × 10^-6^ s; peak power per pixel (irradiation intensity), 3 × 10^5^ W/cm^2^; overall dose per pixel (dose in mJ), 1.5 mJ/cm^2^.

#### UVA laser, 405 nm

Diode laser; laser power, 50 mW; irradiated area, 25.8 × 10^-8^ cm^2^; irradiation time, 4 s; resolution for image acquisition, 512 × 512; line average, 96; pixel size, 60.06 × 60.06 nm; image size, 30.74 × 30.74 μm; total number of irradiated pixels, 24,776; irradiation time per pixel, 161 × 10^-6^ s; peak power per pixel (irradiation intensity), 1.9 × 10^5^ W/cm^2^; overall dose per pixel (radiation dose in mJ), 1.2 mJ/cm^2^.

#### UVA lamp

Lamp from UVC Servis, Czech Republic; model GESP-15 15 W (UVA 330 to 400 nm wavelength, maximal efficiency 365 nm; geometry of irradiation, vertically downward; distance from the sample, 10 cm; irradiated area, 9.2 cm^2^ (cultivation plate area); irradiation time, 15 min.; dose, 0.828 J/cm^2^; cells were seeded at 6.5 × 10^4^ cells/cm^2^ and irradiated 24 h after seeding without BrdU pre-sensitization.

#### γ-rays

Source, ^60^Co from Chisostat, Chirana, Prague; cells were cultivated on 22.1 cm^2^ cell cultivation plates at a density of 6.5 × 10^4^ cells/cm^2^ and irradiated by 5 Gy of γ-rays (total dose); a maximum of four Petri dishes were irradiated on a rotation platform; the irradiation time was 2 to 3 min and the distance of the radiation source from the samples was 110 cm.

### Immunofluorescence, Western blots, and immunoprecipitation

Cells on microscope slides were fixed in 4% formaldehyde for 10 min at room temperature, permeabilized sequentially in 0.2% Triton X-100 for 10 min and 0.1% saponin (Sigma-Aldrich, CZ) for 12 min, and washed twice in PBS for 15 min. Slides were blocked with 1% BSA dissolved in PBS for 1 h, washed for 15 min in PBS, and incubated with the following antibodies: anti-HP1β (#07-333, Upstate, USA or #MAB3448, Merck-Millipore, USA), anti-γH2AX (phospho S139; #ab2893, Abcam, UK), anti-53BP1 (#ab21083, Abcam); anti-UBF1 (H-300; sc-9131, Santa Cruz Biotechnology, USA), anti-RPA194 (#sc-48385, Santa Cruz Biotechnology), anti-TCOF (Treacher Collins syndrome protein, #ab65212, Abcam), and anti-fibrillarin (#ab5821, Abcam). Secondary antibodies used were: Alexa Fluor® 594 Donkey Anti-Mouse IgG (H + L) (A21203), Alexa Fluor® 594 Donkey Anti-Rabbit IgG (H + L) (A21207), Alexa Fluor® 488 Donkey Anti-Mouse IgG (H + L) (A21202), and Alexa Fluor® 488 Donkey Anti-Rabbit IgG (H + L) (A21206) (Molecular Probes®, Life Technologies, Czech Republic).

UVA microirradiation-induced CPDs in iMEFs were investigated using anti-CPD antibody (#NMDND001, COSMO BIO Co., Ltd., Japan) (Figure 2B,C). Methodology was performed according to [31].

Western blotting was performed according to the method of Stixová *et al.* [48]. For Western blot analysis of UBF1, we used anti-UBF1 (#sc9131, Santa Cruz), which detects UBF1 (97 kDa), UBF2 (94 kDa), and GFP-UBF1 (124 kDa) [49]. Apoptotic lamin B fragmentation was examined using anti-lamin B antibody (#sc-6217, Santa Cruz, USA) to determine whether GFP-UBF1 overexpression induced cell death. Anti-HP1β (HP1β: 28 kDa; GFP-HP1β: 55 kDa) (#MAB3448, Merck-Millipore) and anti-H3K9me3 (17 kDa) (#07-442, Merck-Millipore) antibodies were also used.

Immunoprecipitation was performed according to the method of Dawson *et al.* [50]. We used the following antibodies for immunoprecipitation: anti-UBF1 (H-300; sc-9131, Santa Cruz Biotechnology, USA) (Figure 7A) and anti-HP1β (#MAB3448, Merck-Millipore) (Figure 7C). We performed immunoprecipitation with and without EtBr solution (E1510, Sigma-Aldrich, Czech Republic). To eliminate DNA-mediated protein interactions, EtBr was maintained in cell lysates at a concentration of 50 μg/ml during the entire immunoprecipitation process, including washing steps. We used 20 μg of total protein lysate as input. Immunoprecipitation was also performed with cells transfected by plasmid DNA encoding GFP-HP1β-ΔCSD and full-length GFP-HP1β. For Western blotting and immunoprecipitation analyses, we performed three biological replicates.

### RNA interference

RNA interference (RNAi) for HP1β was performed using Lipofectamine RNAiMax transfection reagent (#13778-075, Invitrogen), siRNA (HP1β) (#sc-35588, Santa Cruz Biotechnology, Inc., Germany), control siRNA (#sc-36869, Santa Cruz Biotechnology, Inc.), and Opti-Mem reduced serum medium (#31985047, Invitrogen). The RNAi procedure was performed according to the manufacturer’s instructions for Lipofectamine RNAiMax reagent.

### Analysis of GFP-UBF1 fluorescence intensity in nucleoli and non-nucleolar genomic regions after UVA irradiation

We selected three or four sub-regions with the same areas within irradiated and non-irradiated nucleolar regions to analyze GFP-UBF1 fluorescence intensity (Figure 3B,C). We analyzed fluorescence intensity in nucleoli and the nucleoplasm (that is, outside the nucleoli). The background fluorescence intensity (non-irradiated nucleoli or nucleoplasm) was subtracted from irradiated ROI fluorescence intensity. In some cases, this yielded negative values, owing to bleaching of the fluorochrome in irradiated regions, which reduced the level of fluorescence. This was observed primarily in nucleoli characterized by very slow UBF1 diffusion ( [51]; see Figure 3C for an example of negative values).

### FRET

The FRET technique was performed using Leica SP5 X and Leica SPX8 confocal microscopes and the FRET mode of LEICA LAS AF software (version 2.1.2). To determine protein interactions, we used the FRET acceptor photobleaching technique [52]. Proteins were labeled with mCherry and GFP (or by Alexa 594 and Alexa 488). Using these fluorochromes, FRET was optimized for well-known interacting partners, such as 53BP1 and p53, which yielded a FRET efficiency of approximately 40% (Figure 4A). To study protein interactions, we selected ROIs and measured donor fluorescence intensity before the bleaching step. We performed bleaching with 100% laser power for the acceptor. Finally, we measured fluorescence intensity after acceptor photobleaching, and calculated FRET efficiency using LEICA LAS AF software. We also analyzed UBF1 (visualized by Alexa 594) interaction with DNA stained by TO-PRO-3 iodide (642/661) (Life Technologies, Czech Republic).

### RNA isolation and qRT-PCR

Total RNA was isolated from NIH 3T3 cells with the RNeasy Mini Kit (#74104; Qiagen) according to the manufacturer’s instructions. In most experiments, cells for RNA extraction were collected from 60.1-cm^2^ Petri dishes. cDNAs were generated by reverse transcription of RNAs using Superscript II reverse transcriptase (Invitrogen) and oligo(dT) primers (Sigma, Czech Republic). PCR was carried out on an Applied Biosystems 7300 cycler instrument using the Fast Start SYBR Green Master mix (Roche). Two sets of *HP1β* primers amplifying the conserved Cbx1 domain were designed, based on the cDNA sequence (GenBank # BC055290). The first pair was *mHP1β* (42,60, the number corresponds to the first nucleotide of the forward and reverse primer) forward: 5′-AGAAGAAGAGGAAGAGGAA-3′ and *mHP1β* (176,193) reverse: 5′-CAATAAGGTCAGGGCAAT-3′. The second pair was *mHP1β* (175,192) forward: 5′-GATTGCCCTGACCTTATT-3′ and *mHP1β* (281,298) reverse: 5′-GTTTGCTTTCCTCTCCTT-3′. Primers for the reference *β-actin* and *glyceraldehyde dehydrogenase* (*GAPDH*) genes were designed according to published sequences [53]. Reactions (15 μl) were carried out in the same PCR cycle in the 96-well plate format. The amplification conditions were as follows: initial denaturation at 95°C for 3 min followed by 40 cycles of 15 s at 95°C, 20 s at 57°C, and 30 s at 72°C. Cycle threshold (Ct) values ranged from 17 to 21. Expression levels were calculated using Microsoft Excel. The samples involved three technical replicates, and expression data were obtained for two biological replicates. Normalized expression levels were calculated from Ct values using the formulas:


The fold change was calculated as: 

The results are shown as fold change.

## Electronic supplementary material

Additional file 1: Figure S1: Patterns of selected nucleolar protein accumulation at UVA-induced DNA lesions. Levels of **(A)** RPA194 (red), **(B)** TCOF (red), and **(C)** fibrillarin (red) in UVA-irradiated ROIs (yellow) in 3T3 cells stably expressing GFP-HP1β (green). Cell nuclei were visualized under transmission light and by DAPI (blue) after fixation in 4% formaldehyde. For each event, 20 to 30 nuclei were analyzed in three independent experiments. IF, immunofluorescence; LCI, live-cell image. (TIFF 13 MB)

Additional file 2: Figure S2: **(A)** Visualization of the following endogenous nucleolar proteins: **(a)** UBF1/2, **(b)** RPA194, **(c)** TCOF, and **(d)** fibrillarin in control non-irradiated 3T3 cells stably expressing GFP-HP1β (green). Cells were analyzed by immunofluorescence and confocal microscopy. For each event, 20 to 30 nuclei were analyzed in three independent experiments. **(B)** Western blot analysis shows levels of recombinant GFP-UBF1, endogenous UBF1, UBF2, and lamin B. Data were normalized to total protein levels. IF, immunofluorescence; iMEF, immortalized mouse embryonic fibroblasts. (TIFF 16 MB)

## Abbreviations

ATM: ataxia telangiectasia mutated
ATR: ataxia telangiectasia and Rad3-related protein
BrdU: 5-bromo-2′-deoxy-uridine
BSA: bovine serum albumin
CD: chromodomain
CPD: cyclobutane pyrimidine dimer
CSD: chromo shadow domain
Ct: cycle threshold
DAPI: 4',6-diamidino-2-phenylindole, DDR, DNA-damage response
DMEM: Dulbecco’s modified Eagle’s medium
DSBs: double-strand breaks
EtBr: ethidium bromide
FBS: fetal bovine serum
FRET: Förster resonance energy transfer
Fucci: fluorescent ubiquitination-based cell-cycle indicator
*GAPDH*: glyceraldehyde dehydrogenase gene
GFP: green fluorescence protein
HP1: heterochromatin protein 1
iMEFs: immortalized mouse embryonic fibroblasts
MEFs: mouse embryonic fibroblasts
PBS: phosphate-buffered saline
PCR: polymerase chain reaction
qRT-PCR: quantitative real-time polymerase chain reaction
RFP: red fluorescence protein
RNA pol I: RNA polymerase I
ROI: Region of interest
RNAi: RNA interference
RPA: replication-related protein A
TCR: transcription-coupled repair
UBF1: upstream binding factor 1
UVA: ultraviolet A.
