# Supplementary figures and images for: HP1β-dependent recruitment of UBF1 to irradiated chromatin occurs simultaneously with CPDs
Source: Epigenetics Chromatin. 2014 Dec 30;7:39. doi: 10.1186/1756-8935-7-39 (PMC4293114; doi:10.1186/1756-8935-7-39)

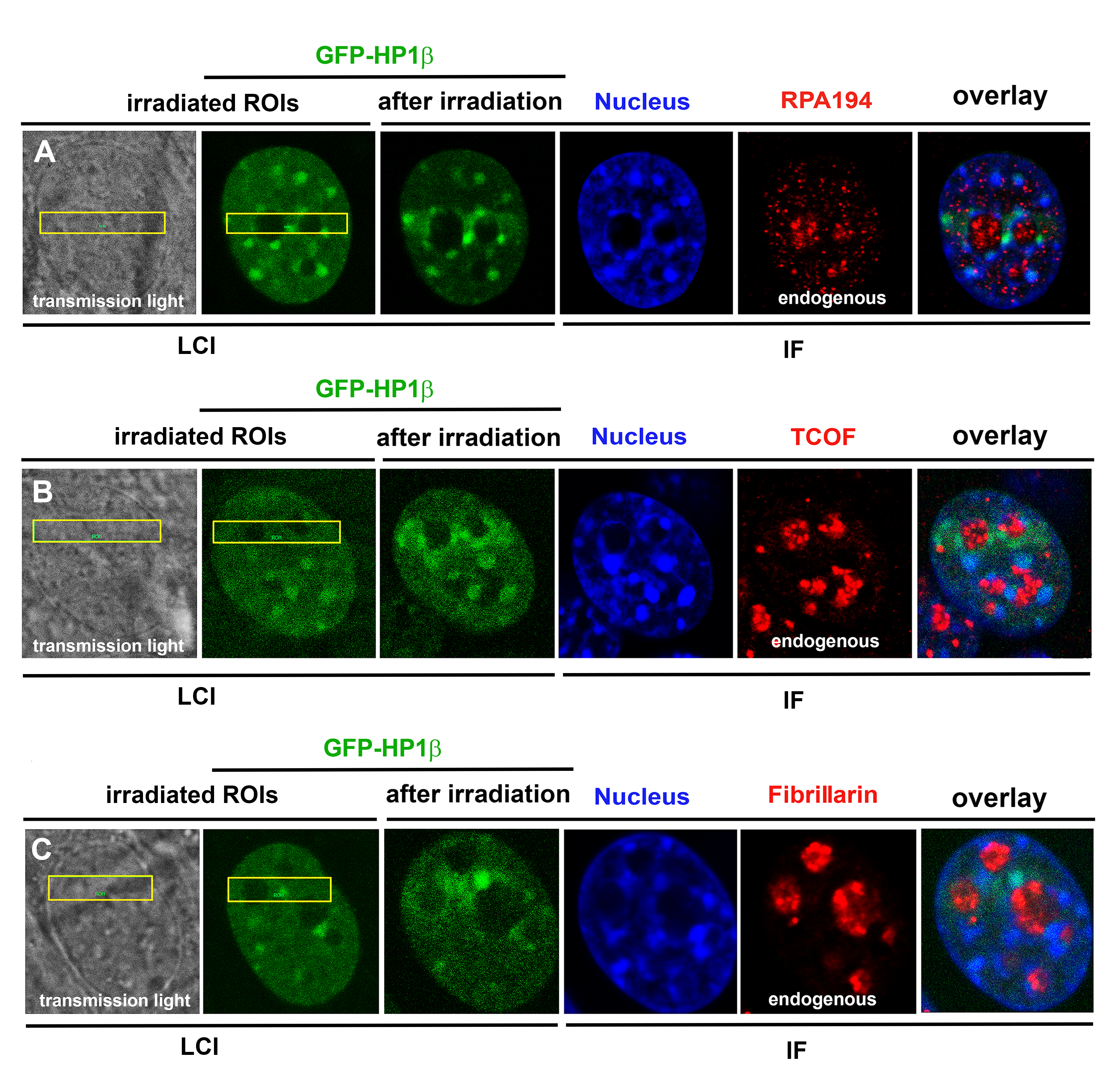

Supplement: Supplementary file 1 — Additional file 1: Figure S1: Patterns of selected nucleolar protein accumulation at UVA-induced DNA lesions. Levels of (A) RPA194 (red), (B) TCOF (red), and (C) fibrillarin (red) in UVA-irradiated ROIs (yellow) in 3T3 cells stably expressing GFP-HP1β (green). Cell nuclei were visualized under transmission light and by DAPI (blue) after fixation in 4% formaldehyde. For each event, 20 to 30 nuclei were analyzed in three independent experiments. IF, immunofluorescence; LCI, live-cell image. (TIFF 13 MB) [file 13072_2014_343_MOESM1_ESM.tiff]

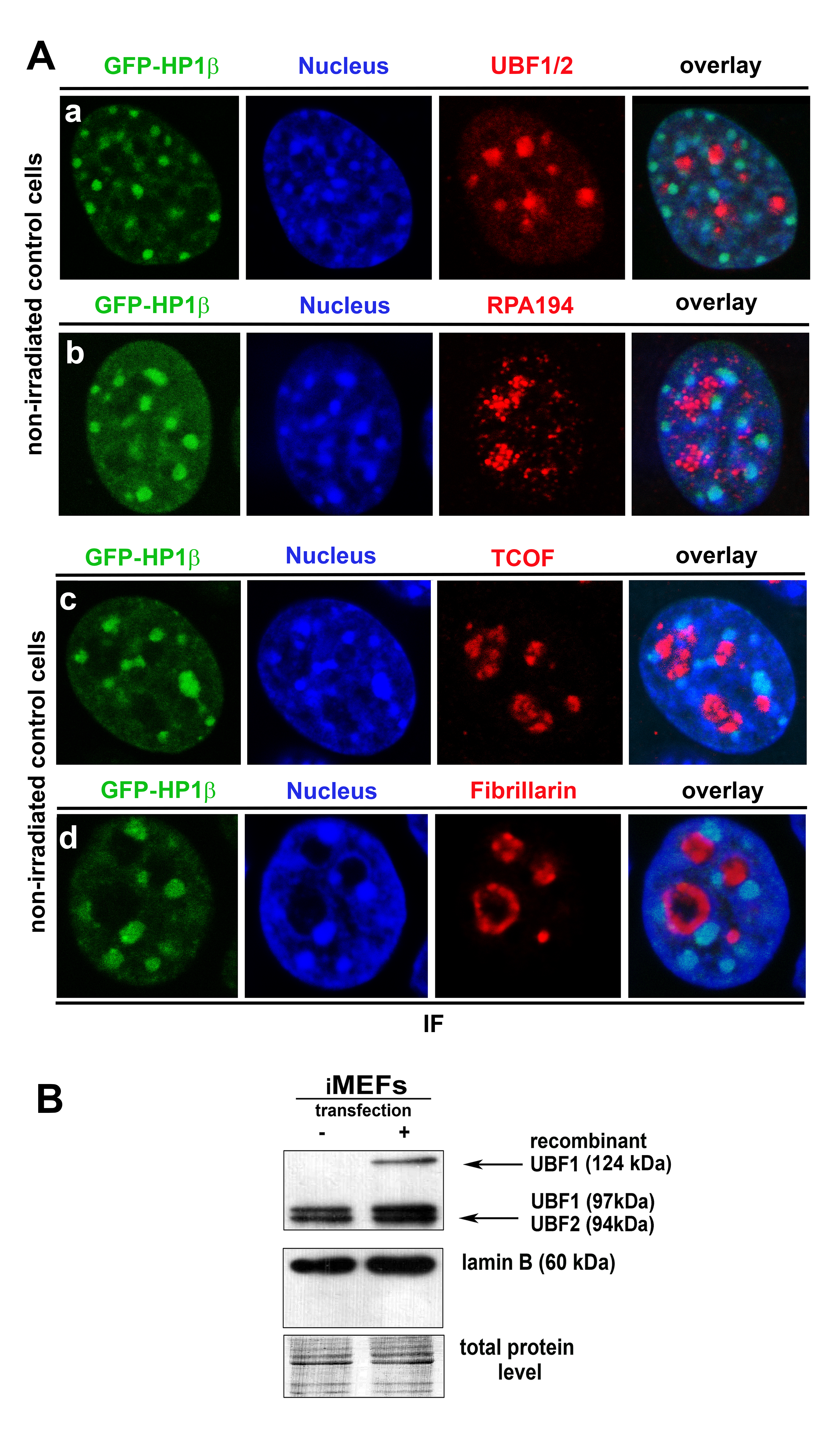

Supplement: Supplementary file 2 — Additional file 2: Figure S2: (A) Visualization of the following endogenous nucleolar proteins: (a) UBF1/2, (b) RPA194, (c) TCOF, and (d) fibrillarin in control non-irradiated 3T3 cells stably expressing GFP-HP1β (green). Cells were analyzed by immunofluorescence and confocal microscopy. For each event, 20 to 30 nuclei were analyzed in three independent experiments. (B) Western blot analysis shows levels of recombinant GFP-UBF1, endogenous UBF1, UBF2, and lamin B. Data were normalized to total protein levels. IF, immunofluorescence; iMEF, immortalized mouse embryonic fibroblasts. (TIFF 16 MB) [file 13072_2014_343_MOESM2_ESM.tiff]
